# Heart-type fatty-acid-binding protein (FABP3) is a lysophosphatidic acid-binding protein in human coronary artery endothelial cells

**DOI:** 10.1016/j.fob.2014.10.014

**Published:** 2014-10-31

**Authors:** Ryoko Tsukahara, Hisao Haniu, Yoshikazu Matsuda, Tamotsu Tsukahara

**Affiliations:** a Department of Hematology and Immunology, Kanazawa Medical University, 1-1 Daigaku, Uchinada, Ishikawa 920-0293, Japan; b Endowed Research Division of Human Welfare Sciences, Ochanomizu University, 2-1-1 Ohtsuka, Bunkyo-ku, Tokyo 112-8610, Japan; c Science and Education Center, Ochanomizu University, 2-1-1 Ohtsuka, Bunkyo-ku, Tokyo 112-861, Japan; d Institute for Biomedical Sciences, Shinshu University Interdisciplinary Cluster for Cutting Edge Research, 3-1-1 Asahi, Matsumoto, Nagano 390-8621, Japan; e Clinical Pharmacology Educational Center, Nihon Pharmaceutical University, Ina-machi, Saitama 362-0806, Japan

**Keywords:** FABP3, fatty-acid-binding protein 3, muscle and heart, HCAECs, human coronary artery endothelial cells, LPA, lysophosphatidic acid, LPC, lysophosphatidylcholine, PPARγ, peroxisome proliferator-activated receptor gamma, siRNA, small interfering RNA, FABP3, Lysophosphatidic acid, HCAEC, Peroxisome proliferator-activated receptor gamma

## Abstract

Fatty-acid-binding protein 3, muscle and heart (FABP3), also known as heart-type FABP, is a member of the family of intracellular lipid-binding proteins. It is a small cytoplasmic protein with a molecular mass of about 15 kDa. FABPs are known to be carrier proteins for transporting fatty acids and other lipophilic substances from the cytoplasm to the nucleus, where these lipids are released to a group of nuclear receptors such as peroxisome proliferator-activated receptors (PPARs). In this study, using lysophosphatidic acid (LPA)-coated agarose beads, we have identified FABP3 as an LPA carrier protein in human coronary artery endothelial cells (HCAECs). Administration of LPA to HCAECs resulted in a dose-dependent increase in PPARγ activation. Furthermore, the LPA-induced PPARγ activation was abolished when the FABP3 expression was reduced using small interfering RNA (siRNA). We further show that the nuclear fraction of control HCAECs contained a significant amount of exogenously added LPA, whereas FABP3 siRNA-transfected HCAECs had a decreased level of LPA in the nucleus. Taken together, these results suggest that FABP3 governs the transcriptional activities of LPA by targeting them to cognate PPARγ in the nucleus.

## Highlights

• We have identified FABP3 as an LPA carrier protein in human coronary artery endothelial cells (HCAECs).
• Administration of LPA to HCAECs resulted in a dose-dependent increase in PPARγ activation.
• LPA-induced PPARγ activation was abolished when the FABP3 expression was reduced using small interfering RNA (siRNA).
• FABP3 governs the transcriptional activities of LPA by targeting them to cognate PPARγ in the nucleus.

## 1 Introduction

Fatty-acid-binding protein 3, muscle and heart (FABP3) is one of nine known cytosolic FABPs ranging in size from 14 to 15 kDa. It is most ubiquitously expressed in heart, skeletal muscle, and other tissues [1,2]. The protein is involved in maintaining an energy supply to the heart [3] and other body parts [4], as well as in regulating the intramuscular fat content [5] and improving insulin sensitivity [6].

Shan et al. reported *FABP3* works as being the response gene of peroxisome proliferator-activated receptor gamma (PPARγ) [7]. PPARγ is a member of the nuclear hormone receptor superfamily, many members of which function as lipid-activated transcription factors [8]. These gene regulatory factors have a direct impact on human diseases such as atherosclerosis, obesity, diabetes, and cancer [9–12]. Gene transcription by PPARγ has been shown to be regulated by ligands such as retinoic acid and long-chain fatty acids [13], which bind in cells to members of the family of intracellular lipid binding proteins, including FABPs. Published X-ray crystal structures of FABPs have revealed that the entrance to the ligand-binding pockets is flanked by two *α*-helices that appear to limit access to the binding site [14]. In this study, we show that lysophosphatidic acid (LPA)-bound FABP3 can be displaced by LPA but not by lysophosphatidylcholine (LPC). Previous studies have provided evidence that LPA is an intracellular agonist for PPARγ, and might enter cells to activate the PPARγ. In the inactivated state, PPARγ is believed to exist as complexes, bound with corepressor proteins such as nuclear receptor corepressor and silencing mediator of retinoid [8]. In this state, PPARγ may have a cytoplasmic rather than a nuclear location. Upon ligand activation, PPARγ dissociates from the corepressor and recruits coactivators [15]. However, the important question that remains to be answered is how lipids like the poorly water-soluble LPA reach the nucleus to bind to PPARγ and initiate transcriptional activity. Here, we present evidence that FABP3 functions as an intracellular carrier for LPA.

## 2 Materials and methods

### 2.1 Reagents and antibodies

LPA-coated agarose beads (L-6101) and control beads (P-B000) were purchased from Echelon Biosciences (Salt Lake City, UT, USA). The tissue culture media were obtained from Nacalai Tesque (Kyoto, Japan). Rabbit polyclonal antibody against cardiac FABP (ab45966) was purchased from Abcam (Cambridge, UK), and mouse polyclonal antibody against β-actin (sc-47778) was purchased from Santa Cruz Biotechnology (Santa Cruz, CA, USA).

### 2.2 Cell culture

Primary HCAECs (Lonza, Walkersville, MD, USA) were propagated in endothelial cell growth medium (EGM-2) containing 5% fetal bovine serum, manufacturer-recommended supplemental growth factors (EGM-2 BulletKit), antibiotics, and antimycotics. All assays were performed using cells taken between passages 3 and 12 at 60–80% confluence, and were repeated at least three times in duplicate or triplicate.

### 2.3 Pull-down assay with LPA beads

HCAECs (1.0 × 10^6^ cells) were lysed on ice for 30 min in a cell lysis buffer (20 mM Tris–HCl [pH 7.4], 10% [v/v] glycerol, 100 mM NaCl, 1% [v/v] Triton X-100, 1:100 protease inhibitor cocktail [Sigma–Aldrich], and 1 mM dithiothreitol) and then centrifuged at 16,000×*g* for 20 min at 4 °C. The resulting cytosolic extracts were assayed for pull-down. One milliliter of cytoplasmic extraction from HCAECs was incubated with 20 μL of the LPA beads at 4 °C for 1 h in lysis buffer. The beads were washed five times with a wash buffer (20 mM 2-(*N*-morpholino)ethanesulfonic acid [pH 7.4] and 150 mM NaCl), and the proteins were eluted with 30 μM LPA in the wash buffer. Samples were separated on a 5–20% sodium dodecyl sulfate–polyacrylamide gel electrophoresis (SDS–PAGE) gel and silver stained (WAKO, Tokyo, Japan).

### 2.4 In-gel digestion and protein identification by matrix-assisted laser desorption/ionization-time-of-flight mass spectrometry

In-gel digestion of gel bands was performed as described previously [16]. Briefly, protein spots excised from the gel were destained with 100 mM ammonium bicarbonate in 50% acetonitrile. The gel pieces were dried and digested with sequencing-grade modified trypsin (Promega). The peptide solution was recovered, and residual peptides were extracted by shaking the solution with 5% trifluoroacetic acid (TFA) in 50% acetonitrile. The combined solutions were concentrated using a lyophilizer. The tryptic peptides, which were dissolved in 0.1% TFA, were desalted with Zip-Tip (Millipore, Billerica, MA, USA) according to the manufacturer’s instructions, mixed with an equal volume of a matrix solution (10 mg/mL α-cyano-4-hydroxycinnamic acid in 50% acetonitrile/0.1% TFA), and applied to a target plate. MS/MS analyses were performed using the AB SCIEX TOF/TOF™ 5800 System (AB SCIEX, Foster City, CA, USA). Protein identification was performed with the ProteinPilot™ software (AB Sciex) using the UniProt database.

### 2.5 Western blotting

The cytoplasmic and nuclear extracts were isolated using NE-PER nuclear and cytoplasmic extraction reagents (Pierce, Rockford, IL, USA). The protein extracts were then separated on 5–20% SDS–PAGE gels (e-PAGEL; ATTO, Tokyo, Japan) and electrotransferred to Immobilon-P membranes (Millipore). The membranes were blocked in Block Ace (DS Parma Biomedical Co. Ltd., Osaka, Japan) for 1 h and then incubated with a primary antibody in Tris-buffered saline-Tween 20 with 5% Block Ace for 12 h at 4 °C. Bands were visualized with EzWestLumi plus (ATTO).

### 2.6 Analysis of LPA (18:1) by liquid chromatography–tandem mass spectrometry

Total lipids were extracted from whole cells by the use of butanol. The samples were diluted to 1:1000 in methanol/water (95:5, v/v) containing 5 mM ammonium formate, and the amount of LPA (18:1) was measured by liquid chromatography–tandem mass spectrometry (LC–MS/MS). The LC–MS/MS was performed using a quadrupole–linear ion trap hybrid MS, 5500 QTRAP (Applied Biosystems/AB Sciex, Concord, ON, Canada), with an Agilent 1200 LC system combined with an autosampler (Agilent Technologies, Wilmington, DE, USA). The LPA (18:1) was separated on an Inertsil® ODS-3 column (150 mm × 2.1 mm; with 3 μm silica particles; GL Sciences Inc., Tokyo, Japan), which was eluted with methanol/water (95:5, v/v) containing 5 mM ammonium formate at a flow rate of 0.2 mL/min. Aliquots (10 μL) of the test solutions were applied to the mass spectrometer for analysis. The LPA (18:1) was analyzed by multiple reaction monitoring in the negative-ion mode, with Q3 (product ion) set at *m*/*z* 281.2 in combination with the deprotonated molecular ion as Q1 (*m*/*z* 435.16). The relative amount of LPA (18:1) was calculated from the ratios of the peak areas using Analyst® software (AB SCIEX).

### 2.7 Quantitative real-time polymerase chain reaction (PCR) analysis

Total RNA was prepared from HCAECs cells using NucleoSpin® RNA II (Takara, Shiga, Japan). Then, 0.5 μg of total RNA was used for the subsequent synthesis of cDNA using the ReverTra Ace qPCR RT Kit (Toyobo, Osaka, Japan) as recommended by the manufacturer. mRNA levels were quantified by using an ECO Real-Time PCR system (Illumina, Inc., San Diego, CA, USA) and the SYBR Green Realtime PCR Master Mix Plus (Toyobo) with the following primers: CD36, 5′-GGCTGTGACCGGAACTGTG-3′ (F) and 5′-AGGTCTCCAACTGGCATTAGAA-3′ (R), and CYP27A1, 5′-CGGCAACGGAGCTTAGAGG-3′ (F) and 5′-GGCATAGCCTTGAACGAACAG-3′ (R) Primers from a human housekeeping gene primer set (Takara) were used as the internal control; the most suitable housekeeping genes were determined according to the manufacturer’s instructions. All PCRs were performed in 10-μL volumes using 48-well PCR plates (Illumina, Hayward, CA, USA). The cycling conditions were 95 °C for 10 min (polymerase activation) followed by 40 cycles of 95 °C for 15 s, 55 °C for 15 s, and 72 °C for 30 s. The relative mRNA quantification was calculated using the arithmetic formula 2^−ΔΔCq^, where ΔCq is the difference between the threshold cycle of a given target cDNA and an endogenous reference cDNA.

## 3 Results

**Fig. 1:**
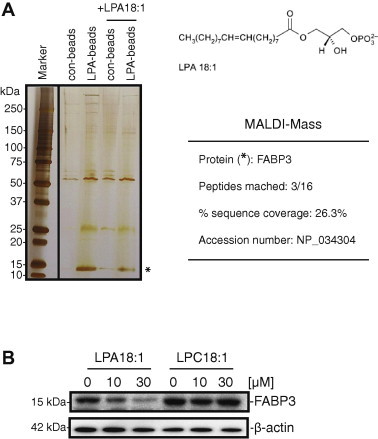
Silver-stained polyacrylamide gel (4–20%) revealing proteins retained by lysophosphatidic acid (LPA) beads. (A) Samples of 1 mL (0.5 mg) of cytosolic proteins were mixed with either control beads or LPA beads. Proteins retained by the beads were eluted with 30 μM LPA and separated by SDS–PAGE. Molecular mass markers are shown on the left. The separated protein bands were visualized by silver staining. The protein band (^∗^) was excised from the gel, digested with trypsin, and identified by mass fingerprinting. The number of peptides, percentage of sequence coverage, and the accession number for the protein are given on the right side. (B) Specificity of heart-type fatty-acid-binding protein (FABP3) binding to LPA. The human coronary artery endothelial cell lysates were preincubated with 0, 10, or 30 μM LPA (18:1) or LPC (18:1) before incubation with LPA beads. Supernatants were analyzed by immunoblotting, using an antibody against FABP3. Incubation with an anti-β-actin antibody was used as a protein-loading control.

**Fig. 2:**
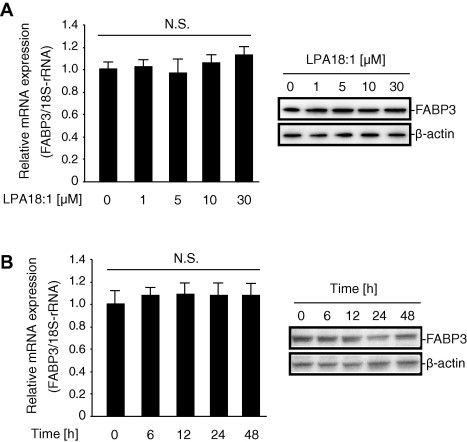
Lysophosphatidic acid (LPA) is not involved in heart-type fatty-acid-binding protein (FABP3) up-regulation in human coronary artery endothelial cells (HCAECs). (A) Real-time PCR measurement of FABP3 mRNA and protein expression in HCAECs. Cells were treated with vehicle (dimethyl sulfoxide) or LPA for 20 h. PCR was performed using specific primers for FABP3. The relative FABP3 levels were normalized to 18S rRNA and are expressed as the mean ± SEM (*n* = 3). Protein levels were analyzed by SDS–PAGE and visualized with the enhanced chemiluminescence reagent. Each lane was loaded with 20 μg of whole-cell lysate. β-Actin was used as the loading control. (B) Cells were treated with vehicle (dimethyl sulfoxide), or LPA for 0, 6, 12, 24, and 48 h. PCR was performed using specific primers for FABP3. The relative FABP3 levels were normalized to 18S rRNA and are expressed as the mean ± SEM (*n* = 3). Protein levels were analyzed by SDS–PAGE and visualized with the enhanced chemiluminescence reagent. Each lane was loaded with 20 μg of whole-cell lysate. β-Actin was used as the loading control.

**Fig. 3:**
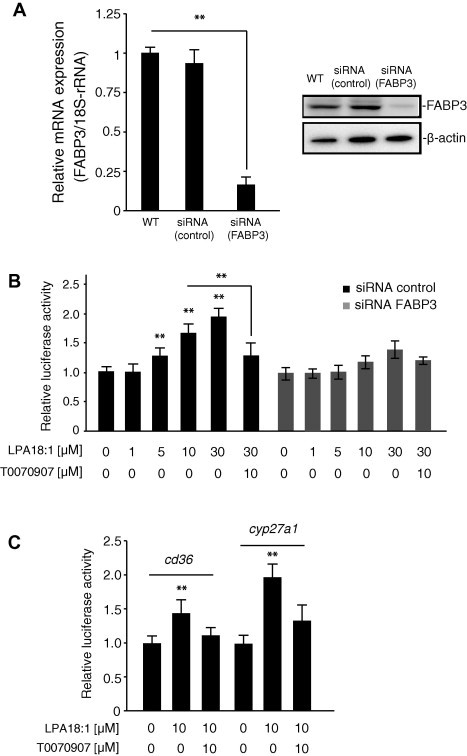
(A) The effect of small interfering RNA on heart-type fatty-acid-binding protein (FABP3) mRNA expression in human coronary artery endothelial cells (HCAECs). The efficiency of FABP3 knockdown was calculated to be 75% by real-time quantitative RT-PCR. Data are presented as the mean ± SEM (*n* = 3). Protein levels were analyzed by SDS–PAGE and visualized with the enhanced chemiluminescence reagent. Each lane was loaded with 20 μg of whole-cell lysate. β-Actin was used as the loading control. (B) Effect of lysophosphatidic acid (LPA) on reporter activation in FABP3-knocked-down HCAECs. FABP3-knocked-down cells were transiently transfected with a pGL3-PPRE-acyl-CoA oxidase luciferase reporter vector. The cells were treated with 1–30 μM LPA for 20 h. Luciferase activity was normalized to *Renilla* luciferase activity. The synthetic peroxisome proliferator-activated receptor gamma antagonist T0070907 (10 μM) was used as the positive control. Data are expressed as the mean ± SEM (*n* = 4), ^∗∗^*P* < 0.01. (C) Induction of LPA-induced expression of PPARγ-regulated genes by in HCAEC. Cells were exposed to a 10 μM LPA or 10 μM T0070907 for 20 h, and RNA was isolated. mRNA levels for the PPARγ upregulated (Cd36, and Cyp27a1) gene targets were determined by real-time quantitative RT-PCR. Relative mRNA abundance ±SEM; *n* = 3; representative experiment shown.

Using affinity chromatography with LPA beads, we successfully captured a potential target protein (15 kDa) from the HCAECs (Fig. 1A). After extensive washing, the 15-kDa protein was excised from the gel, trypsin-digested, and analyzed by peptide mass fingerprinting with MALDI-TOF-MS. Tandem mass spectrometry profiles identifying the FABP3 protein are shown in Fig. 1A. To obtain direct proof of the interaction of LPA with FABP3, the LPA beads were incubated with HCAECs extracts in the presence or absence of 0–30 μM LPA or LPC on ice. The modified FABP3 was isolated from the free LPA or LPC, and then subjected to SDS–PAGE and western blotting against FABP3 antibody. As shown in Fig. 1B, the LPA-bead-bound FABP3 decreased dose-dependently in the presence of excess LPA, but not LPC. This result suggests that LPA interacts with the binding within the FABP3. Next, to determine the role of LPA in regulating FABP3 expression, we examined the effect of LPA on FABP3 expression in HCAECs. As shown in Fig. 2A and 2B, stimulation with LPA (0–30 μM) did not up-regulate the FABP3 mRNA and protein expressions. We then verified whether the protein involved in the LPA-mediated PPARγ activation was indeed FABP3. As shown in Fig. 3A, the knockdown of FABP3 expression in HCAECs using small interfering RNA (siRNA) was effective, as evidenced by real-time polymerase chain reaction (PCR) analysis and western blot analysis using an anti-FABP3 antibody. Real-time PCR analysis showed that FABP3 mRNA in siRNA-transfected cells was knocked down by 75–80% compared with the expression in control cells. As shown in Fig. 3B, the knockdown of FABP3 in HCAECs markedly decreased the ability of the LPA to induce PPARγ-mediated transactivation in a dose-dependent manner. Furthermore, we selected two PPAR target genes and monitored their transcriptional regulation in HCAEC treated with LPA for 20 h. LPA stimulation activated the expression of *Cd36*, and *Cyp27a1* by 1.5- to 2-fold (Fig. 3C).

**Fig. 4:**
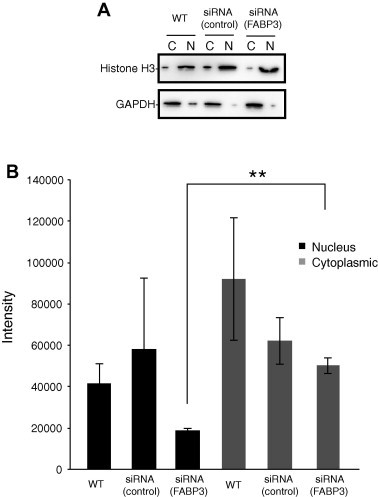
Liquid chromatography–electrospray ionization-mass spectrometry (LC–ESI-MS) quantification of lysophosphatidic acid (LPA; 18:1) in the nuclear fraction of human coronary artery endothelial cells. (A) The nuclear and cytosolic extracts (20 μg each) were analyzed by western blotting using specific antibodies against histone-H3 (nuclear marker) and glyceraldehyde 3-phosphate dehydrogenase (cytoplasmic marker). (B) The samples were diluted to 1:1000 in methanol/water (95:5, v/v) containing 5 mM ammonium formate, and the amount of LPA (18:1) was measured by LC–MS/MS. LC–MS/MS was performed using a quadrupole–linear ion trap hybrid MS, 5500 QTRAP system. Data are expressed as the intensity; *n* = 3.

These results suggest that FABP3 enhances the activity of PPARγ. We then examined the amount of LPA present in the HCAECs. LPA was exogenously added to cells at a concentration of 10 μM, and LPA uptake was determined by LC–MS/MS. As shown in Fig. 4B, interestingly, the nuclear fraction of the FABP3-knocked-down HCAECs had significantly less nuclear LPA than that of the control cells.

## 4 Discussion

Relatively few intracellular binding partners for LPA have been recognized. Previous studies have identified some candidate proteins, including C-terminal-binding protein/brefeldin A-dependent ADP ribosylated substrate [17], liver fatty-acid-binding protein [18], and gelsolin [19]. LPA and alkyl-LPA are also ligands for PPARγ [20,21]. Here, the isolation and purification of LPA-binding proteins from HCAECs were coupled to their identification by proteomics technique. As a result, we provided evidence that FABP3 is distributed from the cytosol to the nucleus in response to LPA-mediated PPARγ activation. It is becoming increasingly apparent that liver specific FABPs act as solubilizing intracellular shuttles for fatty acid ligands to the nucleus, where the ligand is released to its target PPARs [22]. The low water solubility of LPA in the hydrophilic cytosol is its diffusional barrier. To obviate this, intracellular proteins such as FABPs orchestrate the movement of fatty acids inside cell. Our study suggests that FABP3 is ubiquitously expressed in the HCAECs and may therefore serve as intracellular carriers for LPA. We found that LPA is rapidly and efficiently delivered to the nucleus. Such a carrier function is expected to be more efficient than the free diffusion of LPA across the cytosol of endocytic vesicles. The function of FABP3 as an LPA carrier was revealed by the siRNA knockdown experiment. Cells transfected with FABP3 siRNA showed a relatively slow nuclear transport of LPA compared with the wild-type control cells. Furthermore, the reporter gene assay suggested that FABP3 governs the transcriptional activities of LPA by targeting them to cognate PPARγ in the nucleus. FABP3 shuttles LPA to the nucleus, wherein they activate PPARγ. This hypothesis is supported by a recent report that FABP5 mediated the nuclear translocation of retinoic acid and enhanced its activation of PPARs [23]. In conclusion, FABP3 represents the first protein known to transport LPA from the cytosol to the nucleus in human coronary artery endothelial cells.

## 5 Conflict of interest

The authors declare that there is no conflict of interest regarding the publication of this article.
